# Bufothionine induced the mitochondria-mediated apoptosis in H_22_ liver tumor and acute liver injury

**DOI:** 10.1186/s13020-015-0033-1

**Published:** 2015-03-14

**Authors:** Rui-Fang Xie, Zhi-Cheng Li, Pei-Pei Chen, Xin Zhou

**Affiliations:** Department of Pharmacy, Long hua Hospital Affiliated to Shanghai University of Traditional Chinese Medicine, South Wan Ping Road No.725, Shanghai, 200032 China; Department of Surgery, Shanghai Pu Dong Hospital Affiliated to Fu Dan University, Shanghai, 201200 China

## Abstract

**Background:**

Bufothionine is an alkaloid in Cinobufacini (*Huachansu*). This study aims to investigate the effects of bufothionine on liver tumors and acute liver injury.

**Methods:**

In the hepatoprotective experiment, fifty rats were randomly divided into five groups (n = 10): normal saline group, model group, compound glycyrrhizin injection (9.14 mL/kg); cinobufacini injection (3.42 mL/kg) (InjA) and bufothionine (9.77 mL/kg) (BufoA) group. Liver weight indices were recorded to judge the degree of liver swelling, hematoxylin and eosin (H&E) staining of liver tissues was carried out to observe liver histological morphology injury and biochemical indicators including aspartate aminotransferase (AST); alanine aminotransferase (ALT); alkaline phosphatase (ALP); and total bilirubin (TBIL) were determined by modular auto-analyzer. In anti-tumor experiment, H_22_-tumor-bearing mice were randomly divided into five groups (n = 10): normal saline group, model group, cinobufacini injection (InjB) (5.14 mL/kg), bufothionine (8.02 mL/kg) (BufoB) and 5-fluorouracil (5-Fu) (3.42 mL/kg). Tumors were picked out and determined with vernier calipers. Histological morphology of tumors was observed by H&E staining. In SMMC-7721 cells, expressions of proteins related to mitochondria-mediated apoptosis pathway including Bcl-2, Bax, caspase-3, caspase-9, cyto-c, Bid, and p53 were analyzed by western blotting at low, medium, high concentrations of bufothione (3.62 μg/mL, 18.12 μg/mL,90.62 μg/mL).

**Results:**

Butothionine relieved CCl_4_-induced liver morphology, decreased the level of ALT (*P* =2.46 × 10^-2^) and expressed tendency to decrease other biochemical markers including AST, ALP and TBIL. Butothionine could also promote necrosis of tumor tissue in H_22_-tumor-bearing mice and restrained tumor growth with 65.16% inhibition rate. Its mechanism might relate to up-regulation of p53 (at low, mediate and high concentration, corresponding *P* values were 0.142, 0.0257, 0.0162), caspase-3 (*P* = 0.246, 0.0267 and 0.0236), cyto-c (*P* = 0.276, 0.0343 and 0.0429), Bid (*P* = 0.0125, 0.0395 and 0.0132) and Bax (*P* = 0.563, 0.0492 and 0.0357) in a dose-dependent manner, down-regulation of Bcl-2 expression (*P* = 0.0232, 0.0178 and 0.0464), but had no significant effects on caspase-9 (*P* = 0.253, 0.147 and 0.287).

**Conclusion:**

Bufothionine induced the proteins for the mitochondria-mediated apoptosis that inhibits liver tumors and protects the liver against acute injury.

## Background

Cinobufacini (*Huachansu*), extracted from the skins and parotid venom glands of the toad *Bufobufo gargarizans* Cantor, is a Chinese medicine for treatment of swelling, pain, and heart failure [1]. In recent years, cinobufacini injection has also been widely used in clinics with or without chemotherapy for various cancers such as lung cancer, liver cancer, prostate cancer and gallbladder cancer with significant effects [2-9]. Bufadienolides are known to be the main active compounds in cinobufacini [10-12] and these compounds have cardiotonic, anesthetic, and antineoplastic effects as well as stimulatory effects on blood pressure and the respiratory system [13]. However, bufadienolides belong to the cardioactive steroids and may cause adverse heart reactions including arrhythmias, ventricular ectopy, sinus bradycardia, atrial arrhythmias, and hyperkalemia [14], which might limit the clinical application of cinobufacini injection [15]. Therefore, it is necessary to investigate the chemical ingredients and effects of cinobufacini injection.

**Figure 1:**
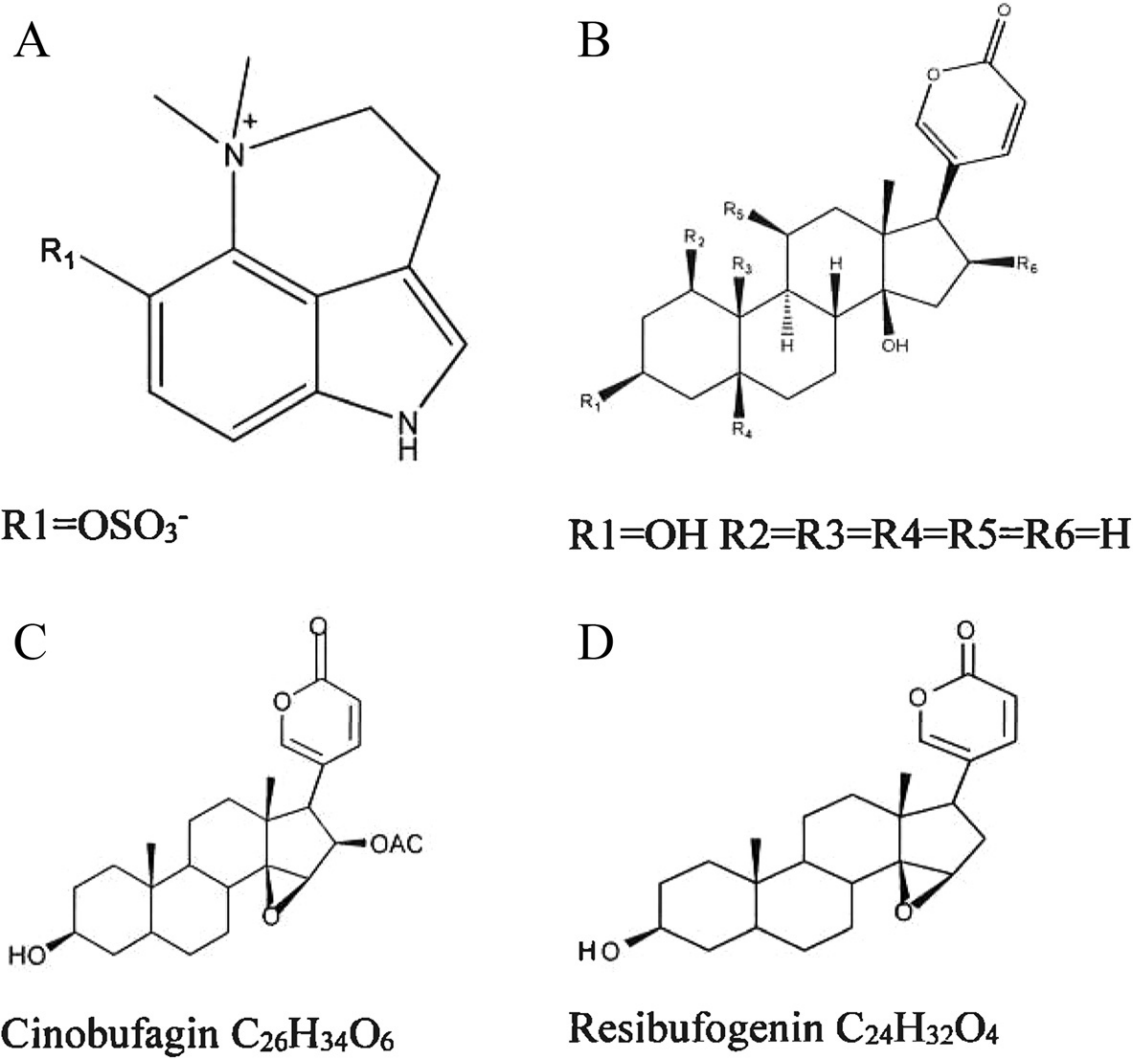
**Structures of bufothionine. (A)**, buffalin **(B)**, cinobufagin **(C)**, and resibufogenin **(D)**.

In our previous work [16], bufothionine was a marker compound in the cinobufacini injection (Figure 1A). Meanwhile, bufadienolides such as cinobufagin and resibufogenin (Figure 1B–D) were under the detection limits. Bufothionine could also inhibit the proliferation of human hepatocellular carcinoma cell lines in a dose- and time-dependent manner, similar to cinobufagin and resibufogenin [16], indicating that bufothionine might be the active component of cinobufacini injection against cancer.

This study aims to investigate the effects of bufothionine on liver tumors and acute liver injury. The effects on protecting liver function were evaluated by rats with acute liver injury induced by carbon tetrachloride (CCl_4_) and the effects on inhibiting liver cancer were assessed by H_22_-tumor-bearing mice. The possible mechanism through the mitochondria-mediated apoptotic signaling pathway was also explored.

## Methods

Bufothionine (Lot: 100201/01–04) and cinobufacini injection (500 mg/mL) (Lots: 090315–2, 090322–2 100614–2) were provided by Jing Chan Pharmaceutical Co. Ltd. China). Glycyrrhizin injection was purchased from Minophagen Pharmaceutical Co. LTD (Japan). CCl_4_ and dimethyl sulfoxide (DMSO) were provided by Sinopharm Chemical Reagent Co. Ltd. (China) and Sangon Biotech (Shanghai) Co. Ltd. (China), respectively.

### Animals

Male Sprague–Dawley rats weighing 170–200 g and male Kunming mice weighing 20–22 g were purchased from Shanghai Laboratory Animals Ltd (China). The animals were maintained at constant temperature (21 − 25°C) and humidity (50 − 70%) on a 12-h/12-h light/dark cycle with free access to food and water. The animals were allowed to adapt to the laboratory environment for at least 7 days prior to the experiments. All procedures were performed according to the guidelines of the National Animal Welfare Law of China. The Experimental Animal Ethical Committee of Shanghai University of Traditional Chinese Medicine care guidelines were complied with to ensure that the mice received human care (Ethics Approval Number: SCXK2012–0002, 2012.1.30).

### Drug preparations

For rats in hepato-protective experiments, 8.4 mg of bufothionine were dissolved in 200 μL of DMSO and diluted with 140 mL normal saline. The final concentration of bufothionine was 58.57 μg/mL. For H_22_-tumor-bearing mice in the anti-hepatocellular carcinoma experiments, 4.5 mg of bufothionine were dissolved in DMSO and diluted with 70 ml normal saline. Obtained concentration of bufothionine was 64.29 μg/mL.

### Acute liver injury test

Fifty rats were randomly divided into 5 groups (10 rats each group) by random digit table generated by SPSS 17.0 software. (SPSS Inc., USA). Both the normal group (Nor) and the model control group (Mod) were treated with intraperitoneal injection of normal saline (including 0.3% DMSO). The rats in the three drug groups were intraperitoneally injected with the corresponding drugs as follows: compound glycyrrhizin injection (9.14 mL/kg) (Gly); cinobufacini injection (3.42 mL/kg) (InjA) and bufothionine (9.77 mL/kg) (BufoA).

All rats were treated once daily at a fixed time (09:00) for 8 days. The weight, appearance and behaviors of each rat including activities, diets and secretions were observed and recorded daily. On day 8 ^th^, CCl_4_ was administered at the dosage of 1.5 mL/kg based on Yokohama *et al.* [17] at 15:00 o’clock to induce acute liver injury in each group except for the normal control group. The rats were fasted with free access to water for 18 h. On the following day (day 9th) at 09:00, all rats were anesthetized with diethylether to collect blood. The biochemical assays of aspartate aminotransferase (AST); alanine aminotransferase (ALT); alkaline phosphatase (ALP); and total bilirubin (TBIL) were performed by a modular autoanalyzer (Roche, Switzerland) [18]. The livers were also collected and weighed. The obtained specimens were fixed in 10% neutral-buffered formalin, dehydrated, and embedded in paraffin for histo-chemical studies.

### Cell lines and culture conditions

Mouse H_22_ hepatoma cells were purchased from American Type Culture Collection (ATCC, USA). The SMMC-7721 cell line was obtained from the Shanghai Institute of Cell Biology, Chinese Academy of Sciences (China). The two cell lines were cultured in RPMI 1640 medium (Gibco, USA) containing 10% heat-inactivated fetal bovine serum (FBS) (Gibco, USA), penicillin (100 U/mL) (Gibco, USA) and 100 mg/L streptomycin (Gibco, USA) at 37°C in a humidified atmosphere of 5% CO_2_ in air. The cells were subcultured every 2–3 days. All cell lines used for experiments remained in the logarithmic growth phase.

### H_22_-tumor-bearing mice test

H_22_ tumor cells were obtained from *in vivo* passages. The stocked murine hepatoma H_22_ cells were subcultured in RPMI 1640 containing 12% FBS and then collected into blank RPMI 1640 culture medium (1 × 10^8^ cells/mL). About 0.2 mL of the cell suspension (approximately 2 × 10^7^ cells) was injected into the abdominal cavity of a mouse. At 5–7 days after the inoculation, ascites occurred in the mice and the fluid was removed using a plastic needle tube (5 mL) under aseptic conditions. About 10–12 mL of fluid was collected from 2–3 mice. The ascites fluid was diluted with an equal amount of normal saline, and centrifuged (Hettich Rotina 420 centrifuge, Germany) at 900 x *g* for 5 min under 4°C. The pellet was mixed with 20 mL of normal saline. Approximately 0.2 mL of the mixture (1 × 10^7^ H_22_ cells) was subcutaneously injected into the right flank of each mouse. Cell mixture (10 mL) could be roughly inoculated into 50 mice.

After 7 days, inoculated mice with tumor sizes of 100–150 mm^3^ were selected and randomly divided into different groups (10–12 mice per group) by random digit tablegenerated by SPSS 17.0 software. Twelve mice without inoculation were characterized as the normal group and injected with normal saline. Among the inoculated groups, one group was injected with normal saline as the model group (Mod) and the other groups were respectively injected intraperitoneally with the following drugs: cinobufacini injection (InjB) (5.14 mL/kg), bufothionine (8.02 mL/kg) (BufoB) and 5-fluorouracil (5-Fu) (3.42 mL/kg). The dosages of drugs for the animals were calculated according to the human dosages recommended on the package inserts. All mice were treated once daily between 09:00 and 10:00 continuously for 10 days except for the 5-Fu group, in which the drug was administered twice a week. The appearances and behaviors were carefully observed. Any abnormal situation such as diarrhea, irritation and emaciation should be recorded. Tumor sizes of the animals were also measured with vernier calipers. Tumor weight indices were expressed as the ratio of tumor weight difference (final tumor weight - initial tumor weight) to body weight. Tumor inhibition rate (D) was obtained with mean tumor indices of drug group divided by mean tumor indices of model group.

When the treatments were over, all animals were deprived of food but allowed free access to water for 16 h. On the following morning, the mice were euthanized for collection of the tumors and spleens, which were immediately placed in 4% buffered paraformaldehyde and fixed overnight. The obtained tumor tissues were dehydrated through an ethanol gradient and embedded in paraffin. Tissue sections (5 μm) were then dewaxed and rehydrated. The sections were stained with hematoxylin and eosin (HE) for observation.

### Western blot analysis

Carcinoma cell line SMMC-7721 cells in the logarithmic growth phase were seeded in 35 mm culture dishes at a density of 3 × 10^5^ cells/mL and incubated for 24 h. Subsequently, the adherent cells were incubated with different concentrations of bufothionine (90.62, 18.12, or 3.62 ng/mL) for 48 h,while the control groups were treated with culture medium containing 10% FBS. The adherent cells were washed twice with ice-cold phosphate-buffered saline (PBS), lysed with RIPA buffer (RIPA buffer/protease inhibitors/EDTA = 100:1:1; Thermo Beyotime, China) for 5–10 min on ice, harvested with a scraper, and centrifuged for 15 min at 14,000 × *g* under 4°C. The resulting supernatants containing the protein fraction were stored at-80°C until analysis.

The protein concentrations were determined with a Bicinchoninic Acid (BCA) Protein Assay Kit (Pierce, USA) according to the manufacturer’s instructions. Afterwards, all protein samples were mixed with Laemmli loading buffer (Beyotime Institute of Biotechnology, China), boiled for 3–5 min, and stored at-20°C until analysis.

Equivalent amounts of proteins were subjected to 8% sodium dodecyl sulfate-polyacrylamide gel electrophoresis and transferred to polyvinyl lidene difluoride membranes (Beyotime Institute of Biotechnology, China). Subsequently, the membranes were blocked with 5% skim milk (Beyotime Institute of Biotechnology) and shaked at 80 r/min for 1 h under room temperature on a shaker and incubated with specific primary antibodies (1:1000 dilution in PBS) against β-actin, Bcl-2, Bax, caspase-3, caspase-9, cyto-c, Bid, and p53 (Santa Cruz Biotechnology Inc., USA) overnight at 4°C. The membranes were washed three times with western cleaning solution (Beyotime Institute of Biotechnology, China), incubated with a horseradish peroxidase-conjugated secondary antibody (1:15,000 dilution in ultrapure water) for 30 min at room temperature in a dark place, and washed three times with western cleaning solution. Finally, immune-reactive proteins were detected by enhanced chemo-luminescence (Odyssey Infrared Imaging System, LI-COR Co. LTD, USA). In this experiment, β-actin was used as an internal control to confirm that the amounts of loaded protein were equal. All experiments were repeated at least three times.

### Statistical analysis

The results were expressed as the mean ± SD. Statistical analyses were performed by one-way analysis of variance (ANOVA) using SPSS 17.0 for Windows software (SPSS Inc., USA). Bonferroni multiple comparison tests were performed for post hoc pairwise comparisons. *P* values less than 0.05 were considered statistically significant. Data from at least three independent experiments were used for statistical analysis. Dose-dependent manner was visually determined.

## Results

### Effects of drugs on acute liver injury

#### Establishment of an acute liver injury model

**Figure 2:**
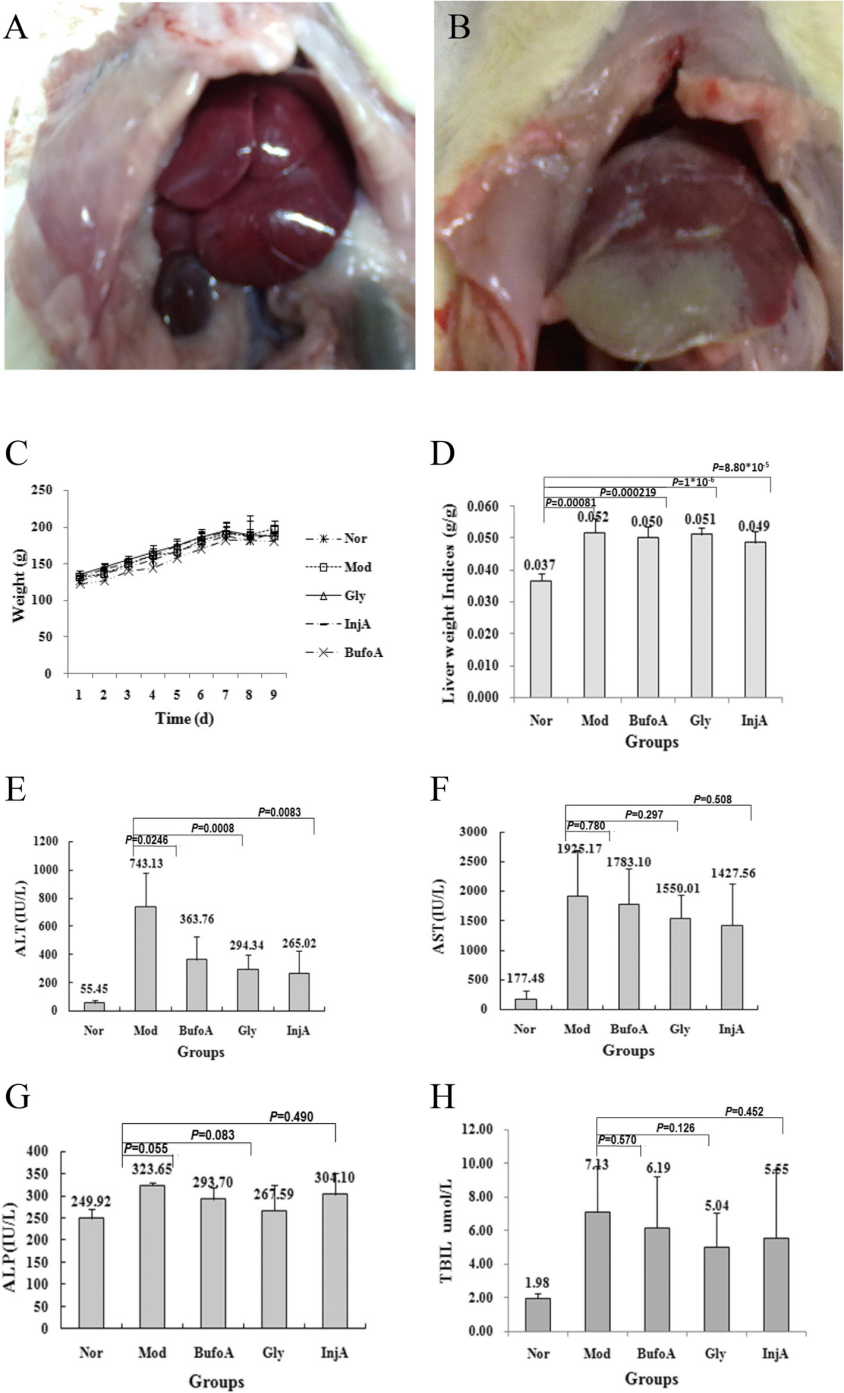
**Effects of drugs on liver morphology, weight and biochemical markers after the CCl**_**4**_ **injury.** All rats were arbitrarily divided into five groups (n = 10): normal group (Nor), model group (Mod), bufothionine group (9.77 mL/kg) (BufoA), glycyrrhizin group (Gly) and cinobufacini injection group (3.42 mL/kg) (InjA). They were treated once daily at a fixed time for 8 d. On the 8th day CCl_4_ (1.5 mL/kg) was administered to induce acute liver injury except in the normal group (**A**: normal rat liver **B**: rat liver model induced by CCl_4_ **)**. Then they fasted with free access to water. On the 9th day, all rats in each group were anaesthetized to collect blood. Weights data during whole experiment were recorded **(C)**. Liver weight indice was calculated as the ratio of liver weight to weight **(D)**. Serum levels of ALT **(E)**, AST **(F)**, ALP **(G)** and TBIL **(H)** were determined. AST: aspartate aminotransferase; ALT: alanine aminotransferase; ALP: alkaline phosphatase; TBIL: total bilirubin. All data were represented mean ± SD (n = 10).

After liver injury was induced by CCl_4_ (1.5 mL/kg), the rats were euthanized and the liver tissues were removed immediately. The normal livers had a bright color with uniform texture (Figure 2A). On the other hand, the injured livers exhibited visible necrosis with a pale color (Figure 2B). The appearance of the rat livers in this trial demonstrated that the acute liver injury model was established.

#### Weight analysis

The weight results showed that the growth rates of the rats were normal during the first 7 days, with an average growth of 8.65 g/d (Figure 2C). However, after acute liver injury induced by CCl_4_, the increments in body weight was arrested on day 8th, indicating that CCl_4_ might contribute to weight loss. There were no significant differences among the groups (*P* = 0.519). These findings indicated that animals were consistent and suitable for subsequent pharmacologic research.

#### Effects of drugs on liver weight indices

Compared with normal group, the rat liver weight indices in the Mod, BufoA, GLy and InjA group were significantly increased (corresponding *P* values respectively 8.10 × 10^-3^, 2.19 × 10^-4^, 1.00 × 10^-4^, 8.80 × 10^-5^) (Figure 2D), indicating the livers might swell after injury.

#### Effects of drugs on liver biochemical markers

Compared with the normal group, ALT, AST, ALP and TBIL values of Mod, BufoA, GLy and InjA group were significantly elevated (corresponding *P* values for ALT were 8.11 × 10^-5^, 1.25 × 10^-3^, 1.18 × 10^-4^ and 1.01 × 10^-2^; for AST, *P* values were 6.66 × 10^-4^, 1.32 × 10^-4^, 2.92 × 10^-6^, 1.93 × 10^-3^; for ALP, *P* values were 1.26 × 10^-4^, 0.01, 0.483, 0.034 and for TBIL, *P* values were 1.02 × 10^-3^, 6.35 × 10^-3^, 3.63 × 10^-3^, 6.04 × 10^-2^) (Figure 2E–H). This suggested that CCl_4_ induced liver injury and resulted in liver dysfunction.

Compared with the model group, ALT levels of BufoA, GLy and InjA group were obviously decreased (corresponding *P* values were 2.46 × 10^-2^, 8.17 × 10^-4^, 8.29 × 10^-3^) (Figure 2E), demonstrating that cinobufacini injection and bufothionine could improve ALT marker. Furthermore, although there was no statistic difference, therapeutic groups also expressed tendency to decrease other biochemical markers including AST, ALP and TBIL (Figure 2F-H).

#### Effects of drugs on liver histological morphology

**Figure 3:**
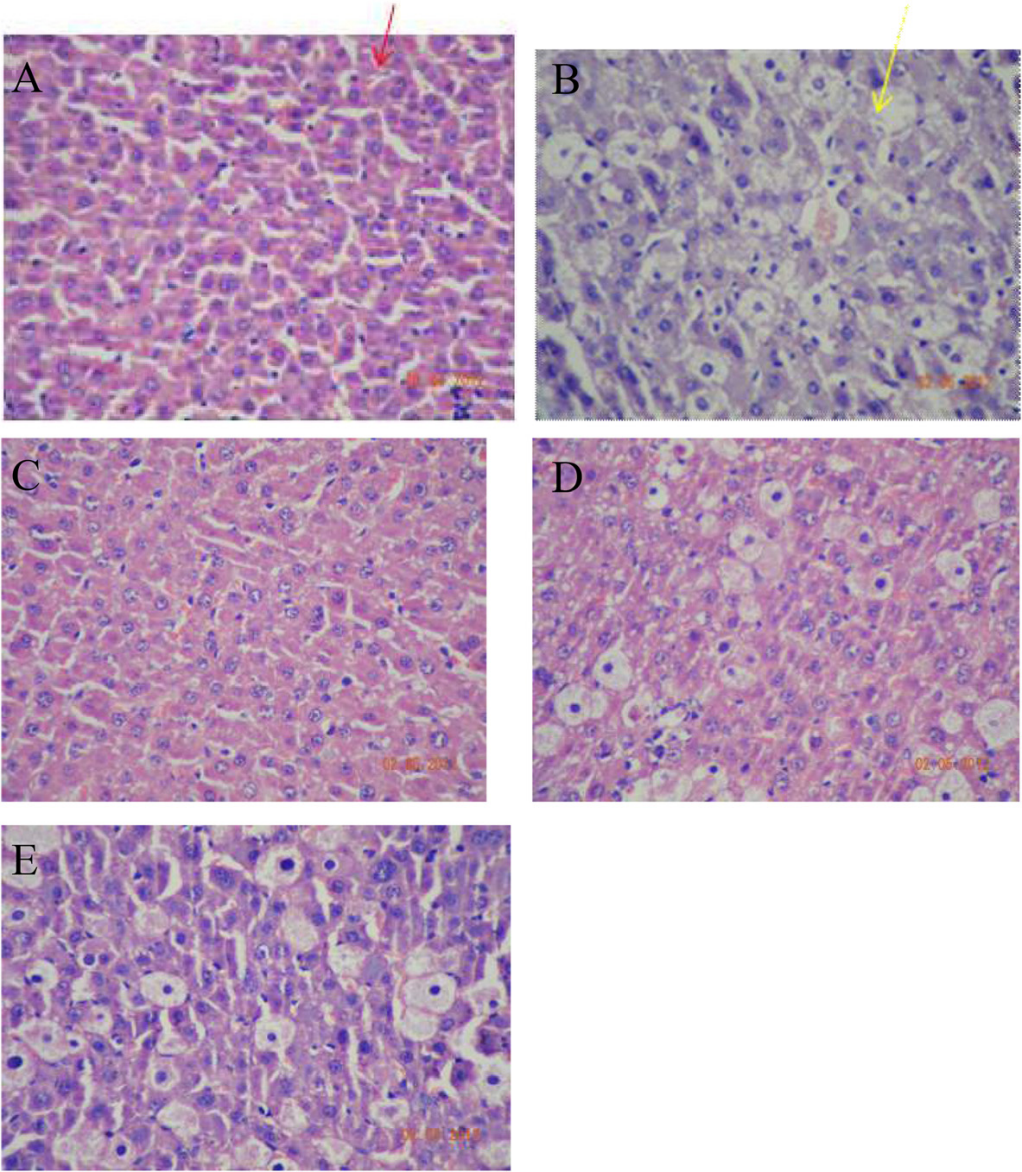
**Effects of drugs on liver histological morphology after CCl**_**4**_ **treatment.** Normal rats **(A)** were administrsted by normal saline without induction by CCl_4_. Other rats were pretreated with normal saline **(B)**, bufothionine (9.77 mL/kg) **(C)**, compound glycyrrhizin injection **(D)**, cinobufacini injection (3.42 mL/kg) **(E)** for 8 days before 1.5 mL/kg CCl_4_ was administered. Hepatic tissue was collected and subjected to hematoxylin and eosin staining. . In normal liver cells cell morphology was normal and the nucleoplasm was uniform (indicated by red arrow). After liver injury, hepatocytes exhibited necrosis and the nucleoplasm was severely contracted (indicated by yellow arrow).

In normal liver cells, the histological morphology and the nucleoplasm was uniform (Figure 3A). In contrast to the normal group, the hepatocytes exhibited necrosis after liver injury and the nucleoplasm was severely contracted (Figure 3B). Through the treatment with drugs, the hepatocyte morphology was improved. The sequence of hepatocyte improvement was as follows: bufoA (Figure 3C) > Gly (Figure 3D) > InjA (Figure 3E).

### Effects of drugs on H_22_-tumor-bearing mice

#### Establishment of an H_22_-tumor-bearing mouse model

**Figure 4:**
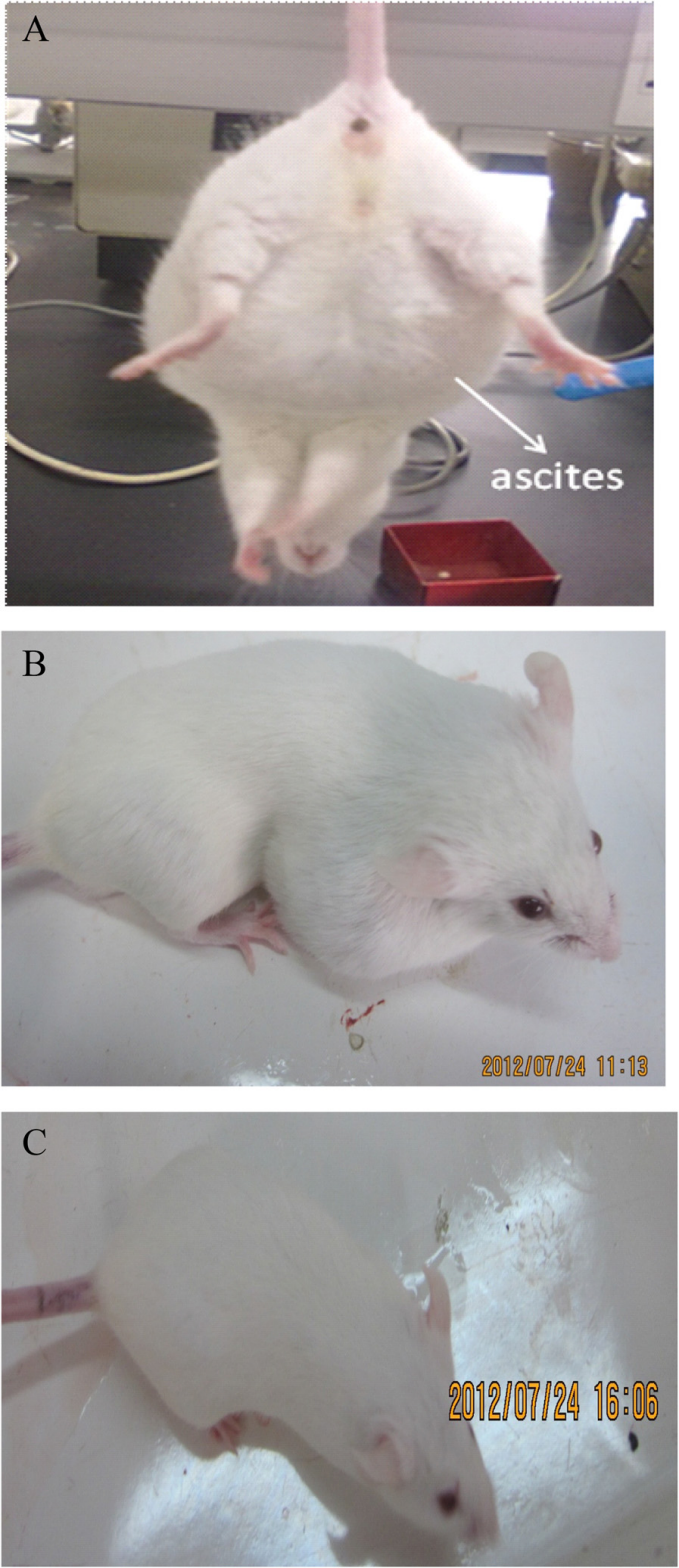
**Establishment of H**_**22**_ **tumor -bearing mice model.** H_22_ cells were injected into the abdominal cavity of a mouse. After several days, ascites occurred **(A)**. Ascites fluids were injected into the right flank of each mouse. After several days, tumor developed under the right flank **(B)**. Mice without inoculation did not develop tumors **(C)**.

At 5–7 days after inoculation of H_22_ cells into the abdominal cavity of a mouse, yellow ascites occurred (Figure 4A). The ascites fluids were extracted, diluted, and centrifuged. The obtained pellets were diluted with normal saline and subcutaneously injected into the right flank of each mouse. After several days, tumors developed under the right flank (Figure 4B), while mice without inoculation did not develop tumors (Figure 4C). These findings indicated that the tumor-bearing mouse model was successfully established.

#### Effects of drugs on weight, spleen weight, and tumor weight

**Figure 5:**
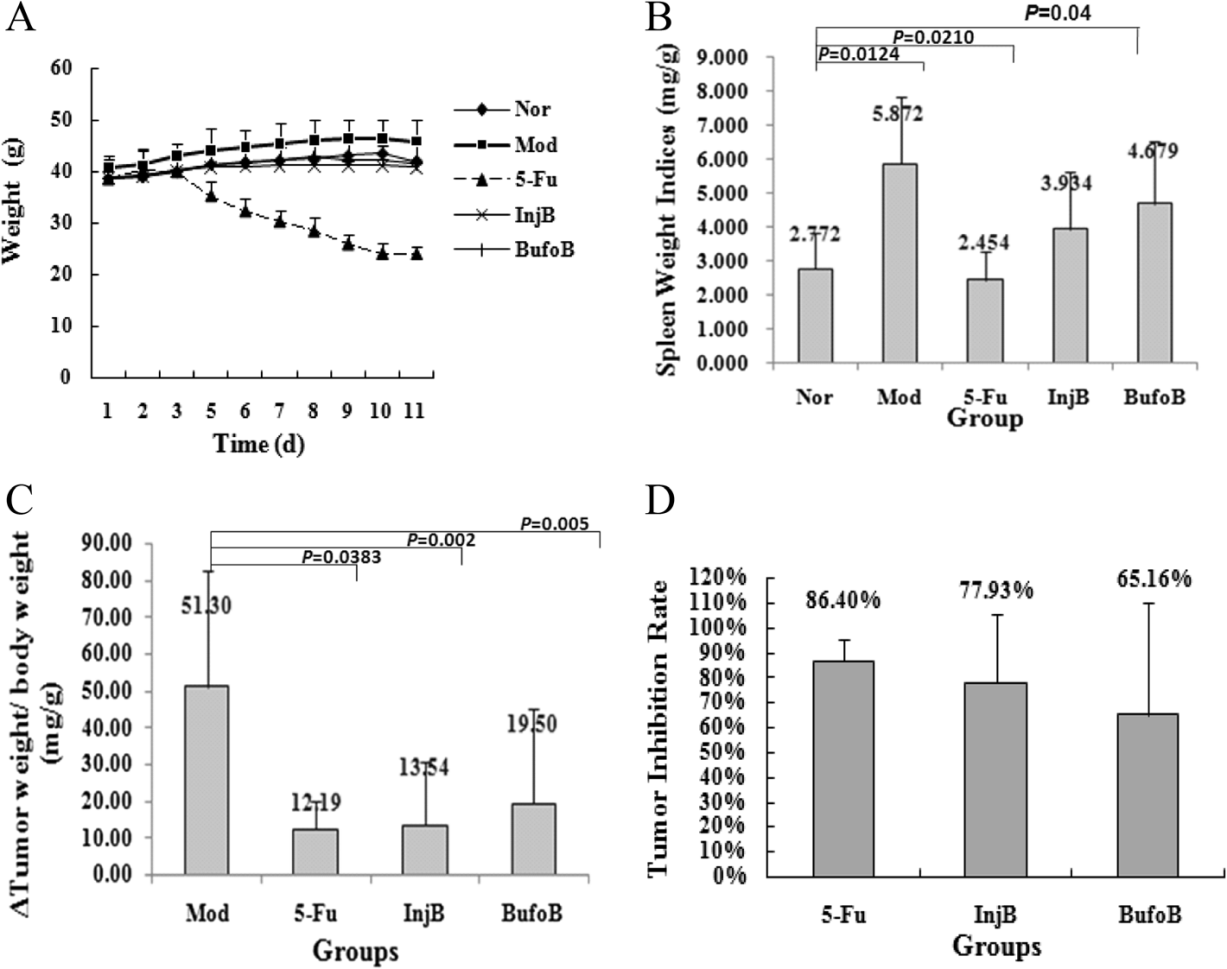
**Effects of drugs on weights. (A)**, spleen weight indices **(B)**, tumor weight indices **(C)** and tumor inhibition rate **(D)** of H_22_ tumor bearing mice. All H_22_ tumor bearing mice were treated with different drugs: normal group (Nor), model group (Mor), bufothionine group (8.02 mL/kg) (BufoB), 5-Fu group (5-Fu), cinobufacini injection group (5.14 mL/kg) (InjB). After ten days, weight **(A)**, spleen weight and tumor weight of different groups were recorded. Spleen weight indices **(B)** were expressed as the ratio of spleen weight to weight. Tumor weight indices **(C)** were expressed as the ratio of tumor weight difference (final tumor weight - initial tumor weight) to body weight. Tumor inhibition rate **(D)** was obtained with mean tumor indices of drug group divided by mean tumor indices of model group. Data were represented as mean ± SD (n = 10).

After inoculation, mice weight increased steadily during the therapeutic period, except for the 5-Fu group. The adverse reactions of 5-Fu chemotherapy were more serious than those of cinobufacini treatment, *e.g.*, diarrhea and weight loss (Figure 5A).

The spleen indices in the therapeutic groups were lower than those in the Mod group (Figure 5B). Among all of the drugs examined, the effect of 5-Fu was the most significant (*P* = 0.0124). BufoB (*P* = 0.04) and InjB group (*P* = 0.0210) also had significant effects on inhibiting spleen swelling.

The tumor indices in the therapeutic groups were lower than those in the Mod group (Figure 5C). Among all of the drugs tested, the effect of 5-Fu was the strongest, with a tumor inhibition rate of 86.40% (Figure 5D). InjB and bufoB group also decreased tumor weight with 77.93% and 65.16% inhibition rates (Figure 5D).

#### Effects of drugs on liver histological morphology

**Figure 6:**
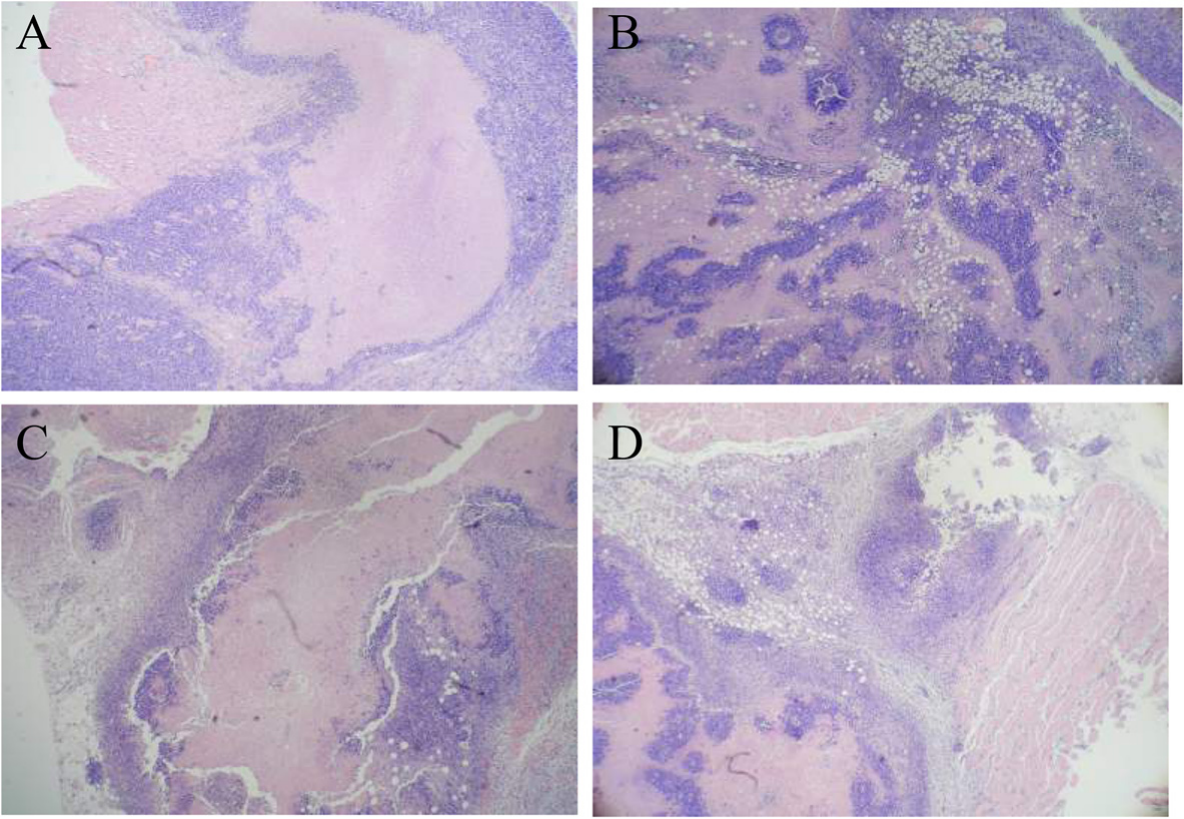
**Effects of drugs on tumor histology for H**_**22**_ **tumor -bearing mice.** Normal saline were administrated to normal rats **(A)**.5-Fu **(B)**, cinobufacini injection (5.14 mL/kg) **(C)** and bufothionine (8.02 mL/kg) **(D)** were respectively administrated to H_22_ tumor-bearing mice. After 10 days, rats were sacrificed and tumors were removed. The tissues were dehydrated, embedded, sliced and stained with HE staining for observation.

In contrast to the Mod group (Figure 6A), the tumor tissues in the therapeutic groups appeared hollow and exhibited necrosis (Figure 6B–D). Among all of the drugs examined, the necrotic areas of tumor tissue in the BufoB group (Figure 6D) were larger than those in all other groups, followed by 5-Fu (Figure 6B) and InjB (Figure 6C).

### Effects of bufothionine on the mitochondria-mediated apoptosis pathway

**Figure 7:**
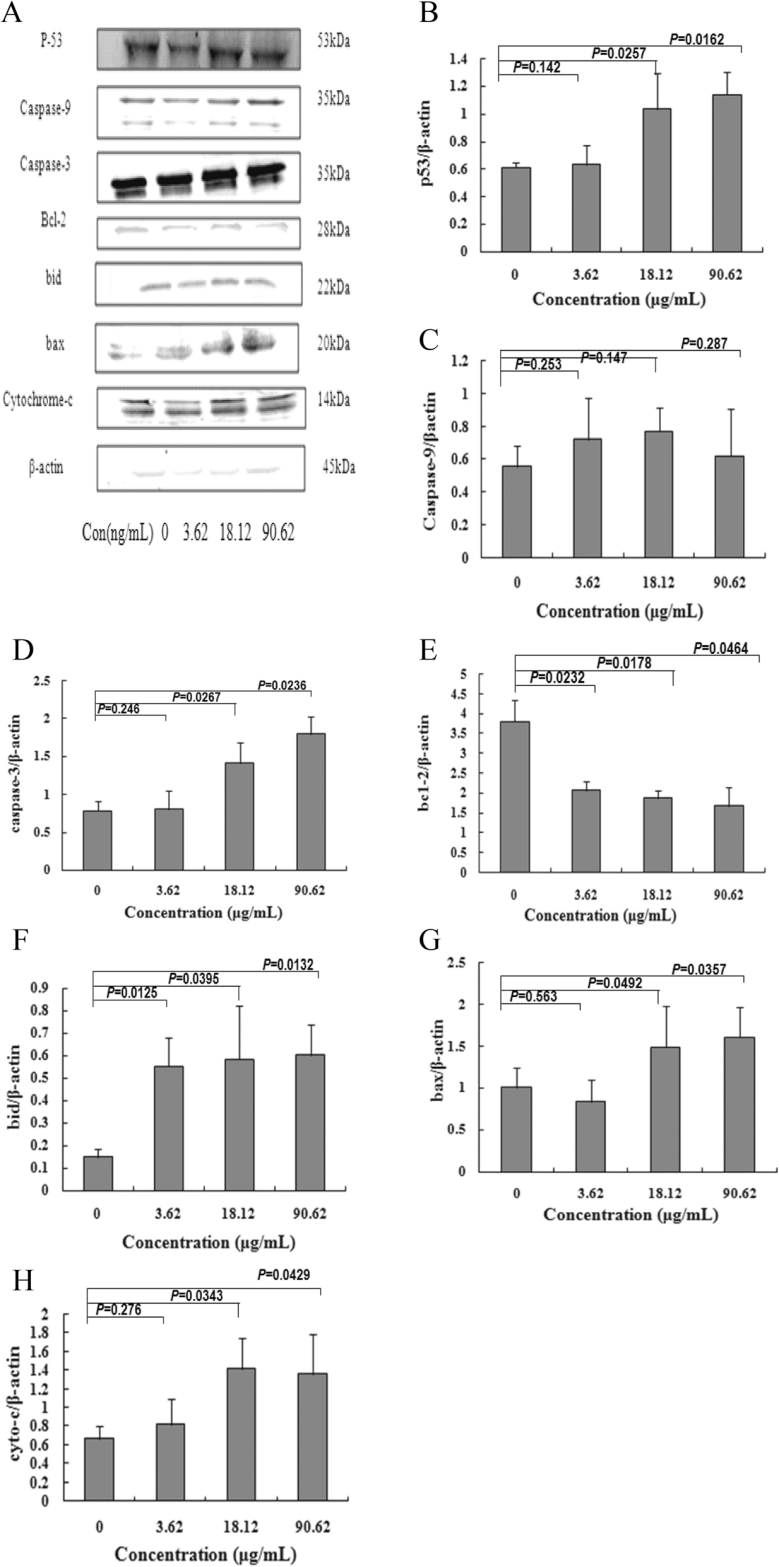
**Effects of bufothionine on the expressions of apoptosis relative proteinsSMMC-7721 cells were treated with different concentration of bufothionine for 48 h (A).** Protein of cells were extracted and protein express of p53 **(B)**, caspase-9 **(C)**, caspase-3 **(D)**, bcl-2 **(E)**, bid **(F)**, bax **(G)** and cyto-c **(H)** was analyzed by Western blot, respectively. All experiments were repeated at least three times. Data were represented as mean ± SD (n = 3).

To explore the involvement of mitochondria-mediated apoptosis induced by bufothionine in SMMC-7721 cells, the expressions of Bcl-2, Bax, caspase-3, caspase-9, Bid, cyto-c, and p53 were measured by western blot analyses (Figure 7). The results revealed that, bufothionine showed a trend toward up-regulating the expressions of p53 (at the low, medium and high concentration corresponding *P* values were respectively 0.142,0.0257 and 0.0162), caspase-3 (*P* = 0.246, 0.0267 and 0.0236), cyto-c (*P* = 0.276, 0.0343 and 0.0429), Bid (*P* = 0.0125, 0.0395 and 0.0132) and Bax (*P* = 0.563, 0.0492 and 0.0357) in a dose-dependent manner, down-regulating the level of Bcl-2 (*P* = 0.0232, 0.0178 and 0.0464), but had no significant effects on caspase-9 (*P* = 0.253, 0.147 and 0.287).

## Discussion

Large numbers of papers about cinobufacini focused on bufodienolides belonging to the cardiotonic steroids [19,20]. To avoid adverse effects of steroids, we focused on the alkaloid bufothionine in this study.

According to clinical practice, cinobufacini injection can relieve liver dysfunctions [6]. For the CCl_4_-induced liver injury model, the dosage of CCl_4_ was the key point to the degree of liver injury, *e.g.*, when the dose was >5 mL/kg body weight, the rats were likely to die within a few hours. After several trials, the dosage of 1.5 mL/kg was considered to be an appropriate dosage. In this model, the results for biochemical markers such as ALT and HE staining showed that bufothionine could improve liver injury.

To our knowledge, CCl_4_ is a well-known hepatotoxic chemical. The CCl_3_^.^ free radicals from CCl_4_ by CYP450 transform, can result in peroxidation of unsaturated fatty acid in the cytoplasm and organelle membranes. The concentrations of calcium ions in cytoplasm are elevated, the cytoskeletons are destroyed, the functions of amino acids are damaged, and the nucleicacids become mutated; and all of the above effects accelerate the death of cells [17]. A possible mechanism for bufothionine to protect liver functions may be associated with its alleviation of injuries caused by free radicals.

Cinobufacini is a Chinese medicine extract for cancer treatment [5,7]. Here, we investigated antitumor effects of its reparation--cinobufacini injection. The H_22_-tumor-bearing mouse model was established according to the literature [21]. It should be noted that the viability of ascites cells was crucial in this trial. At 5–7 days after intraperitoneal injection, the yellow ascites fluids should be collected. If time was delayed, the ascites fluids would become red and the viability of the cells might decrease and result in failure of the experiment. Under this model, bufothionine expressed the effects of inhibit tumor growth.

The antitumor functions of bufadienolides might be asso-ciated with inhibition of cell proliferation [22], induction of apoptosis [23], disruption of the cell cycle [24], promotion of cell differentiation [25], and suppression of angiogenesis [26]. Among these stages, induction of apoptosis was a critical step for bufadienolides. Moreover, the mitochondria-mediated apoptosis pathway was important [19,27].

In the mitochondria-mediated apoptosis pathway, an apoptotic stimulus is followed by release of cyto-c from mitochondria into the cytosol. Following its release, cyto-c forms a complex in the cytoplasm, which then activates procaspase-9 and in turn activates procaspase-3 [28], leading to DNA fragmentation and triggering apoptosis [29]. Bufothionine could promote the release of cyto-c into the cytosol, with up-regulation of its protein expression. Moreover, caspase activity analyses demonstrated that caspase-3 was activated after cinobufacini treatment.

Bufothionine could up-regulate the expression of the pro-apoptotic protein Bax and down-regulate the expression of the anti-apoptotic protein Bcl-2. Members of the Bcl-2 protein family, such as Bax and Bcl-2, are the most prominent players in the mitochondria-mediated apoptosis pathway [30]. Bax is a pro-apoptotic protein [31], while Bcl-2 is an anti-apoptotic protein [32].

The present study also indicated that bufothionine up-regulated the expression of p53. P53 is an important protein in cell apoptosis. Either loss or mutation of p53 leads to gain-of-function toward cell migration, invasion and metastasis [33].

## Conclusion

Bufothionine induced the proteins for the mitochondria-mediated apoptosis that inhibits liver tumors and protects the liver against acute injury.

## Abbreviations

5-Fu: 5-fluorouracil
ALP: Alkaline phosphatase
ALT: Alanine aminotransferase
ANOVA: One-way analysis of variance
AST: Aspartate aminotransferase
BufoA: Bufothionine (9.77 mL/kg)
BufoB: Bufothionine (8.02 mL/kg)
CCl_4_: Carbon tetrachloride
cyto-c: Cytochrome-c
DMSO: Dimethyl sulfoxide
Gly: Compound glycyrrhizin injection
HBV: Hepatitis B virus
H&E: Hematoxylin and eosin
InjA: Cinobufacini injection (3.42 mL/kg)
InjB: Cinobufacini injection (5.14 mL/kg)
Mod: Model control group
Nor: Normal group
PBS: Phosphate buffered solution
PVDF: Polyvinylidenedifluoride
SDS-PAGE: Dodecyl sulfatepolyacrylamide gel electrophoresis
TBIL: Total bilirubin
